# Lurasidone ameliorates doxorubicin-induced chemobrain in male Wistar rats: involvement of the hippocampal and prefrontal cortex CREB/BDNF/Glt-1 axis and glutamate homeostasis

**DOI:** 10.3389/fphar.2026.1901510

**Published:** 2026-09-09

**Authors:** Mohamed Z. Habib, Sherif A. Kamar, Mohamed Atef Elkholy, Yasmin M. Aboul-Ela, Mohamed Mahmoud Abdelrahim Elshaer, Nourhan Moussa, Nouran K. Olama, Sara Elsayed Abdelrahman, Mohammed R. Rabei, Ahmed A. Salama, Esraa M. Elnahas

**Affiliations:** 1 Basic Medical Sciences Department, Faculty of Dentistry, Al-Ahliyya Amman University (AAU), Amman, Jordan; 2 Clinical Pharmacology Department, Faculty of Medicine, Ain Shams University, Cairo, Egypt; 3 Anatomy and Embryology Department, Faculty of Medicine, Ain Shams University, Cairo, Egypt; 4 Biomedical Sciences Department, College of Medicine, Gulf Medical University, Ajman, United Arab Emirates; 5 Histology Department, Faculty of Medicine, Ain Shams University, Cairo, Egypt; 6 Biochemistry and Molecular Biology Department, Faculty of Medicine, Ain Shams University, Cairo, Egypt; 7 Basic Health Sciences Department, College of Applied Medical Sciences, Qassim University, Buraydah, Saudi Arabia; 8 Medical Physiology Department, Faculty of Medicine, Mansoura University, Mansoura, Egypt; 9 College of Dentistry, Gulf Medical University, Ajman, United Arab Emirates; 10 Pediatric Dentistry Department, Faculty of Dentistry, Cairo University, Cairo, Egypt

**Keywords:** astrocyte, chemobrain, doxorubicin, glutamate, glutamate transporter-1, lurasidone

## Abstract

**Introduction:**

Chemotherapy-induced cognitive impairment, commonly referred to as “chemobrain”, represents a frequent and distressing complication of cancer therapy. Doxorubicin (DOX), a widely used chemotherapeutic agent, contributes to these deficits partly through mechanisms involving oxidative stress, inflammatory cascades, and disruption of glutamate homeostasis, but unfortunately, the precise role of altered glutamate transport and neurotrophic signaling remains poorly addressed. Lurasidone (LURA), an atypical antipsychotic, exhibits potent 5-HT7 receptor antagonism and pro-cognitive properties, making it a strong candidate to counteract these deficits. However, its potential to rescue glutamate homeostasis and neuroplasticity pathways in chemobrain has not been explored. This study investigated whether LURA could counteract DOX-induced chemobrain by restoring CREB/BDNF/Glt-1 signaling and re-establishing glutamate homeostasis in the hippocampus and prefrontal cortex.

**Methods:**

Forty-eight male Wistar rats were assigned to control, LURA, DOX, or DOX/LURA groups. Behavioral performance was assessed using the open field, novel object recognition, and Morris water maze tests. Hippocampal and prefrontal tissues were evaluated for protein expression of the CREB/BDNF/Glt-1 pathway, glutamate and GABA levels, oxidative stress indices, and histopathological and immunohistochemical markers of neurodegeneration, apoptosis, and astrocytic activation.

**Results:**

DOX administration resulted in pronounced cognitive decline, oxidative stress, neuroinflammation, neuronal loss, and substantial downregulation of CREB/BDNF/Glt-1 signaling, accompanied by elevated glutamate and reduced GABA levels. LURA co-treatment markedly improved behavioral outcomes, reinstated CREB/BDNF/Glt-1 expression, ameliorated glutamate/GABA balance, and reduced neurodegeneration, caspase-3 activation, and astrocytic reactivity.

**Discussion:**

This study provides the first evidence that lurasidone mitigates doxorubicin-induced chemobrain. This neuroprotective effect is associated with the restoration of glutamate homeostasis and the upregulation of hippocampal and prefrontal CREB/BDNF/Glt-1 signaling, highlighting lurasidone as a promising therapeutic option for chemotherapy-induced cognitive impairment.

## 1 Introduction

Chemobrain, or chemotherapy-induced cognitive impairment (CICI), refers to the deterioration of cognitive functions in cancer patients during or following chemotherapy (Stangler et al., 2025). The prevalence of CICI ranges between 75% during treatment and 35% several months post-treatment (Rovito et al., 2025). The incidence of chemobrain has risen in parallel with improved cancer survival rates and the application of intensive chemotherapy protocols (Godaert and Dramé, 2025). Chemobrain significantly compromises patients’ quality of life, work performance, and psychological wellbeing, making it a major clinical concern. Thus, elucidating its underlying mechanisms and establishing effective preventive/therapeutic interventions are essential for comprehensive treatment strategies for cancer patients.

Doxorubicin (DOX) is utilized in the treatment of a broad spectrum of malignancies, including breast cancer, lymphoma, leukemia, and sarcomas (Johnson-Arbor et al., 2026). It intercalates into DNA with inhibition of topoisomerase II and generation of reactive oxygen species (ROS), which collectively trigger apoptosis in rapidly proliferating tumor cells (McGowan et al., 2017; John et al., 2021; Alshaer et al., 2025). Accumulating evidence indicates that DOX also exerts neurotoxic effects, leading to neurodegeneration and cognitive impairment (Bhatt et al., 2025). DOX-treated animals display cognitive deficits resembling clinical chemobrain, thereby establishing DOX as a rodent model for investigating CICI (John et al., 2021).

Although DOX poorly penetrates the blood-brain barrier (BBB), it can nonetheless elicit substantial neurotoxicity mainly through indirect systemic mechanisms (Bhatt et al., 2025). A key mechanism involves the induction of oxidative stress and systemic inflammation, with the elevation of circulating pro-inflammatory cytokine levels that may cross and disrupt the BBB. This process promotes glial activation and neuroinflammatory response (Keeney et al., 2018; Alhowail and Aldubayan, 2023; Bhatt et al., 2025). Central to this process, altered glutamatergic neurotransmission could represent a pivotal contributor to the neurocognitive deficits associated with DOX exposure; the central DOX-mediated cytokine overproduction, such as the tumor necrosis factor-α (TNF-α), could activate astrocytes, triggering significant glutamate release (Habbas et al., 2015). On the other hand, DOX was shown to decrease the expression of astrocytic glial transport proteins, hence reducing synaptic glutamate clearance (Thomas et al., 2017).

The persistent elevation of synaptic glutamate results in excessive activation of N-methyl-D-aspartate (NMDA) receptors, triggering calcium influx and downstream cascades that promote oxidative damage, mitochondrial dysfunction, and apoptotic neuronal loss—a pathological process referred to as glutamate-induced excitotoxicity (Nicosia et al., 2024). In this regard, astrocytes play crucial neuroprotective roles by clearing glutamate from the synaptic cleft through the excitatory amino acid transporter 2 (EAAT2) in humans and its rodent homolog glutamate transporter-1 (Glt-1), which mediates over 90% of glutamate uptake in the CNS (Sakai et al., 2025; Zhang et al., 2025). Increasing evidence highlights the critical regulatory function of these transporters in maintaining extracellular glutamate homeostasis and modulating synaptic plasticity (Valtcheva and Venance, 2019).

The cAMP response element-binding protein (CREB), particularly in its phosphorylated form (p-CREB), represents an important positive regulator of Glt-1 expression in astrocytes (Pajarillo et al., 2019). This regulatory function is mediated through a crucial crosstalk with brain-derived neurotrophic factor (BDNF). BDNF signaling *via* its tropomyosin receptor kinase B (TrkB) activates downstream pathways such as the extracellular signal-regulated kinase (ERK) that lead to the phosphorylation and activation of CREB (Wang and Mao, 2019). The p-CREB then binds to the promoter regions of target genes, including BDNF itself, forming a positive feedback loop, which also promotes the transcription of Glt-1 (Karki et al., 2014; Zohny et al., 2023). In addition to its roles in regulating glutamatergic neurotransmission, the coordinated CREB/BDNF/Glt-1 signaling cascade has also been shown to mitigate neuronal apoptosis and neurodegeneration, key pathological events involved in chemobrain (Liu et al., 2016; Zohny et al., 2023).

Lurasidone is an atypical antipsychotic with a multimodal mechanism of action involving antagonism of dopamine D_2_ and serotonin and 5-HT_7_ receptors, along with partial agonism at 5-HT_1A_ receptors (Tarazi and Riva, 2013). Studies suggest it has pro-cognitive effects, particularly in attention and working memory, potentially linked to its 5-HT_7_ antagonism and 5-HT_1A_ agonism (Diao et al., 2022; Olivola et al., 2023). At a molecular level, chronic lurasidone administration in rodents has been shown to increase the expression of BDNF and CREB in the hippocampus and the prefrontal cortex, promoting neuronal plasticity (Fumagalli et al., 2012; Luoni et al., 2014; Begni et al., 2024). Moreover, lurasidone has been shown to modulate astrocytic activity and increase the expression of glutamate membrane transporters in chronic mild stress-exposed rats (Luoni et al., 2015).

To the best of our knowledge, this study could be the first to investigate the potential protective effects of lurasidone against doxorubicin-induced cognitive dysfunction while elucidating its modulatory impact on CREB/BDNF signaling, astrocytic Glt-1 expression, and the glutamate/GABA balance, key molecular events implicated in the pathogenesis of CICI.

## 2 Materials and methods

### 2.1 Animals

A total of forty-eight adult male Wistar rats, aged 9 weeks and weighing 180–220 g, were used in this study. The animals were obtained from the animal facility of the Faculty of Pharmacy, Ain Shams University (Cairo, Egypt), and were raised at the Department of Clinical Pharmacology, Faculty of Medicine, Ain Shams University (Cairo, Egypt). Rats were allowed to acclimate to the facility environment for 2 weeks prior to the commencement of experiments. Animals were kept in groups of four per Plexiglass cage (42 × 25 × 15 cm) under controlled laboratory conditions: an ambient temperature of approximately 22 °C–25 °C, a relative humidity of 55% ± 5%, a 12 h light/dark cycle (at 5:00 a.m. - 5:00 p.m.), and good ventilation. Standard pellet food and water were provided *ad libitum*. Cages were cleaned daily throughout the study course. All procedures were conducted in accordance with the EU Directive 2010/63/EU for animal experiments. All efforts were made to decrease animal suffering as well as to reduce the number of animals used. The study was approved by the Research Ethics Committee of the Faculty of Medicine, Ain Shams University (FMASU-REC; approval number: FMASU R293/2025).

### 2.2 Study groups

Rats were randomly divided into four groups of 12 rats:

- Control group: received daily oral gavage with 1% hydroxyethylcellulose in addition to weekly intraperitoneal (i.p.) injection of phosphate buffered saline (PBS) for 4 weeks.
- LURA group: received daily oral gavage with lurasidone (Al Andalous Pharmaceutical Industries, Cairo, Egypt) dissolved in 1% hydroxyethylcellulose at a dose of 3 mg/kg (Calabrese et al., 2020) in addition to weekly i.p. Injection of PBS for 4 weeks.
- DOX group: received daily oral gavage with 1% hydroxyethylcellulose in addition to weekly i.p. Injection of doxorubicin (Hikma Pharmaceuticals, Giza, Egypt) dissolved in PBS at a dose of 2.5 mg/kg/week for 4 weeks (Moretti et al., 2021).
- DOX/LURA group: received daily oral gavage with lurasidone (3 mg/kg) in addition to weekly i.p. Injection of doxorubicin (2.5 mg/kg/week) for 4 weeks. Lurasidone was administered 1 h before doxorubicin administration.

### 2.3 Body weight and behavioral assessments

**FIGURE 1:**
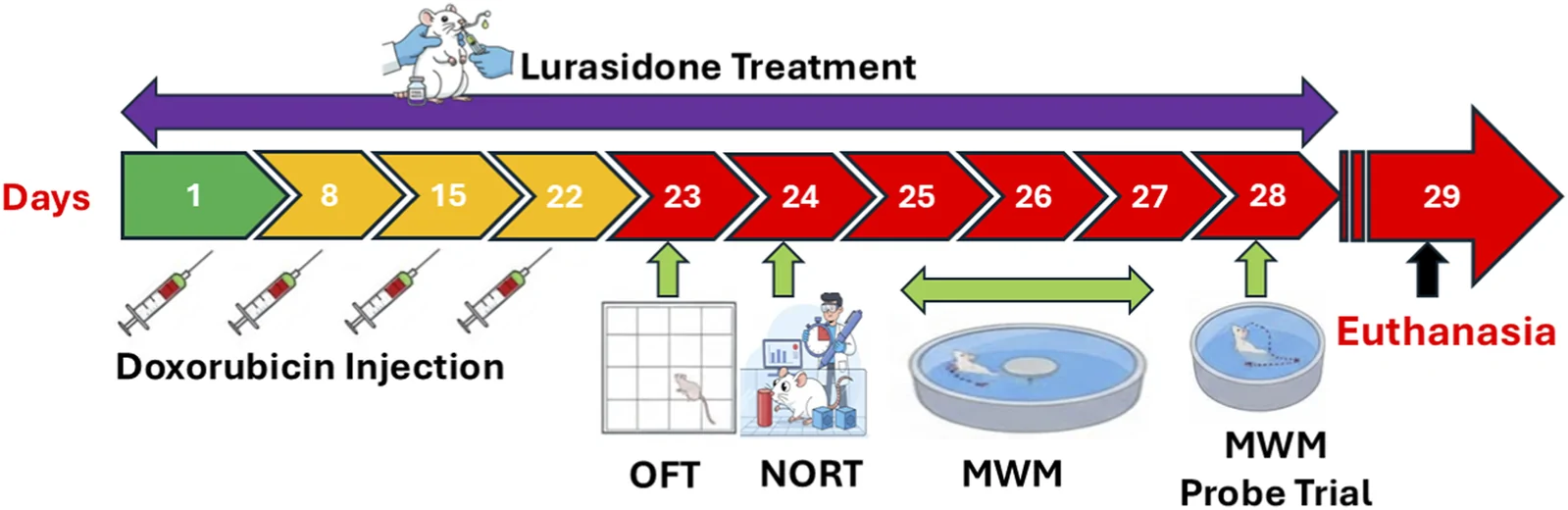
The experimental design of the study. OFT, open field test; NORT, novel object recognition test; MWM, Morris water maze test.

Body weight was measured at the start of the experimental period and weekly thereafter. Behavioral tests were conducted during the final week of the study. Each behavioral test was carried out on a separate day to reduce the effect of sequential testing as follows: open field test on day 23, novel object recognition test on day 24, Morris water maze (MWM) training phase on days 25–27, and finally the MWM probe trial on day 28. For each behavioral test, the rat’s behavior was recorded using a video camera to be analyzed by a well-trained blinded observer. Blinding was also applied during biochemical and histological assessments. Drug doses were administered after completing the behavioral testing on the corresponding day. The timeline of the experimental protocol is illustrated in Figure 1.

#### 2.3.1 Open field test (OFT)

The open field test was performed to evaluate locomotor activity and exploratory behavior in rats. The test was conducted in a square wooden arena measuring 60 × 60 × 45 cm, with the floor divided into equal squares (each 15 × 15 cm). The arena was placed in a quiet and dimly lit room (ambient light intensity: approximately 20 lux) to minimize external disturbances. Prior to testing, all rats were acclimatized to the testing room for at least 30 min to reduce the stress related to environmental novelty. Afterwards, each rat was gently placed in the center of the arena and allowed to explore freely for 5 min. The behavior was recorded by a video camera positioned above the apparatus for subsequent blinded manual analysis by two independent observers. The following parameters were measured: the number of crossed squares entered with all four paws (as an index of the general locomotor activity), the frequency of entries into the central zone (the central four squares) and the time spent in the central zone (as indices of anxiety-related behaviors), and the frequency of rearing (as an index of exploratory activity). Between the testing of individual animals, the apparatus was thoroughly cleaned with 70% ethanol and dried to eliminate olfactory cues (Habib et al., 2026).

#### 2.3.2 Novel object recognition test (NORT)

The NORT was carried out to assess recognition memory and cognitive performance in rats. The test was conducted in a dark quadrangular arena (40 × 40 × 40 cm) made of non-reflective material to minimize visual distraction and was located in a sound-attenuated room. The arena was illuminated by dim, indirect light (approximately 20 lux). Twenty-four hours prior to testing, each rat was acclimatized to the arena for 10 min to reduce anxiety and familiarize it with the environment. The test consisted of two trials: Trial 1 (familiarization phase), which included the placement of two identical objects (water-filled plastic square-shaped white bottles) symmetrically in opposite corners of the arena, approximately 10 cm away from the walls. Each rat was placed gently in the center of the arena, facing away from the objects, and allowed to freely explore for 10 min. Trial 2 (recognition phase), which started after a short retention interval (1 h) during which the rats were returned to their home cages, involved the placement of one familiar object with a novel object of similar size but different shape and color (a blue cylindrical glass). The rat was reintroduced into the arena for 2 min, under the same conditions. The rat’s exploratory behavior was recorded by a video camera positioned above the arena for subsequent blinded manual analysis. Interaction was defined as the rat directing its nose toward an object at a distance of less than or equal to 2 cm or actively touching it with its nose or whiskers. The following parameter was calculated as an index of recognition memory:

$$\text{Novel object recognition index}=\frac{\text{time}\;\text{of}\;\text{interaction}\;\text{with}\;\text{the}\;\text{novel}\;\text{object}}{\text{time}\;\text{of}\;\text{interaction}\;\text{with}\;\text{both}\;\text{objects}}$$

The arena and all objects were thoroughly cleaned with 70% ethanol between sessions to eliminate olfactory cues. All sessions were conducted in a quiet, dimly lit environment, and the objects were securely fixed to prevent displacement during exploration (Raafat et al., 2023).

#### 2.3.3 Morris water maze (MWM)

The MWM test was performed to evaluate the spatial learning and memory in rats. The apparatus consisted of a circular pool (diameter 180 cm, height 60 cm, and visually divided into four equal quadrants) filled with water to a depth of ≈ 40 cm, maintained at a temperature of 22 °C ± 1 °C. A hidden circular platform (10 cm in diameter) was placed 1 cm below the water surface in the center of one of the quadrants (the target quadrant). Distinct visual cues of various shapes and colors were positioned on the walls surrounding the pool to aid spatial orientation. The testing room was sound-attenuated and illuminated by indirect uniform lighting (approximately 30 lux).

Each rat underwent four training trials per day, from different starting positions, for three consecutive days (total of 12 trials). In each trial, the rat was gently placed into the water facing the wall of the pool and allowed 60 s to locate and climb onto the hidden platform. If the animal failed to find the platform within this period, it was gently guided to the platform and allowed to remain there for 15 s. A 1-h interval was maintained between consecutive trials to minimize fatigue and promote memory consolidation. Twenty-four hours following the last training session, a probe trial was conducted to assess memory retention. The platform was removed, and the rat was released into the pool from a novel starting point, facing the wall, and allowed to swim freely for 60 s. All trials were recorded by an overhead digital video camera suspended above the center of the pool, and parameters were manually analyzed by blinded independent observers. The principal outcome measure was the percentage of time spent in the target quadrant, where the platform had previously been located. After each session, the water was refreshed as needed, and the environment was kept consistent throughout testing (Khedr et al., 2024; Magdy et al., 2025).

### 2.4 Euthanasia and sample collection

At the end of the experimental period, animals were anesthetized with urethane (1.2 g/kg, intraperitoneally). Following confirmation of adequate anesthesia, transcardiac perfusion was performed according to the intended analysis. For the biochemical studies (n = 6 rats/group), transcardiac perfusion was carried out with PBS (pH 7.4). The brains were rapidly collected, and the hippocampus and prefrontal cortex were carefully dissected on ice, then stored at −80 °C for subsequent biochemical assays. For the histological studies (n = 6 rats/group), transcardiac perfusion was performed with 10% neutral buffered formalin. The whole brain was then collected and sagittally sectioned into two hemispheres. The tissue specimens were fixed in 10% neutral buffered formalin, followed by dehydration in ascending grades of ethanol, clearing in xylene, and embedding in paraffin wax (Aref et al., 2025).

### 2.5 Biochemical measurements

#### 2.5.1 Gene expression analysis of *Glt-1* and *BDNF* using real-time quantitative polymerase chain reaction (RT-qPCR)

Total RNA was extracted from homogenized brain tissue samples using the Direct-zol™ RNA Miniprep Plus Kit (Cat# R2072; Zymo Research, Irvine, CA, United States), following the manufacturer’s protocol. Reverse transcription and amplification were carried out in a one-step reaction using the SuperScript™ IV One-Step RT-PCR Kit (Cat# 12594100; Thermo Fisher Scientific, Waltham, MA, United States), according to the supplier’s instructions. The expression levels of the target genes, *GLT-1* and *BDNF*, were quantified relative to the housekeeping gene β-actin. The utilized primer sequences are presented in Table 1. The data were obtained as cycle threshold (Ct) values for each gene, and relative expression levels were calculated using the 2^−ΔΔCt^ method as described by Livak and Schmittgen (2001).

**TABLE 1:** Primer sequences of the studied genes.

Gene	Forward primer	Reverse primer	NCBI reference sequence accession no.
*Glt-1*	5′-TGA​CAA​GCG​TGT​GAC​CAG​ATT​CG-3′	5′-GCT​CGC​CAG​AGT​TGC​TGT​AAG​G-3′	NM_017215.3
*BDNF*	5′-ATC​CAC​TGA​GCA​AAG​CCG​AAC-3′	5′-CAG​CCT​TCA​TGC​AAC​CGA​AGT​A-3′	NM_012513.4
*β-actin*	5′-CAG​CTG​AGA​GGG​AAA​TCG​TG-3′	5′-CGT​TGC​CAA​TAG​TGA​TGA​CC-3′	NM_031144.3

Abbreviations: *Glt-1*, Glutamate transporter-1; *BDNF*, Brain-derived neurotrophic factor.

#### 2.5.2 Measurement of CREB, p-CREB, BDNF, Glt-1, glutamate, and GABA protein levels using the enzyme-linked immunosorbent assay (ELISA)

The protein levels of CREB, p-CREB, BDNF, Glt-1, glutamate, and GABA were quantified in the hippocampus and prefrontal cortex using the ELISA technique. The following commercially available rat ELISA kits were utilized: CREB (cat# ELK9706; ELK Biotechnology, Denver, CO, United States), phospho-CREB (Ser133) (cat# DYC2510; Novus Biologicals, Centennial, CO, United States), BDNF (cat# ab213899; Abcam, Cambridge, UK), Glt-1 (cat# ER1968; FineTest, Wuhan, Hubei, China), glutamate (cat# MBS756400; MyBioSource, San Diego, CA, United States), and GABA (cat# MBS269152; MyBioSource, San Diego, CA, United States). All assays were performed in accordance with the manufacturers’ protocols. Absorbance was measured at 450 nm using a microplate reader. The total protein concentration in each sample was measured using the Bradford assay (Bradford, 1976) to allow normalization of ELISA results to the protein content.

#### 2.5.3 Measurement of total antioxidant capacity (TAC), malondialdehyde (MDA), reduced glutathione (GSH), and nitric oxide (NO)

The levels of TAC, MDA, GSH, and NO in the hippocampus and prefrontal cortex were quantified using specific colorimetric assay kits according to the manufacturers’ protocols. TAC was determined using the total antioxidant capacity colorimetric assay kit (cat# EEA022; Thermo Fisher Scientific, Waltham, MA, United States), MDA using the malondialdehyde colorimetric assay kit (cat# AK0954; Sunlong Biotech, Hangzhou, China), GSH using the reduced glutathione colorimetric assay kit (cat# EU2640; FineTest, Wuhan, Hubei, China), and NO using the nitric oxide colorimetric assay kit (cat# E-BC-K035-M; Elabscience, Houston, TX, United States). Absorbance was measured at 520 nm for TAC, 532 nm for MDA, 450 nm for GSH, and 550 nm for NO using a microplate reader. The concentrations of each parameter were calculated from standard calibration curves and expressed relative to tissue protein content.

### 2.6 Histopathological studies

Five µm sagittal sections were cut and stained with hematoxylin and eosin (H&E) and toluidine blue for demonstration of Nissl’s granules. Paraffin sections were also cut on positively charged slides using the avidin-biotin immune peroxidase technique to be immunohistochemically stained for detection of apoptotic neurons using the anti-caspase-3 antibody [EPR18297] (cat# ab184787; Abcam, Cambridge, UK) (1:1,000 dilution) and for astrocytic activity using the anti-GFAP antibody [EPR1034Y] (cat# ab279291; Abcam, Cambridge, UK) (1:500 dilution). Immunohistochemical sections were counterstained with Hematoxylin. Positive control sections of tonsil for caspase three and cerebral cortex for GFAP were stained. Negative control sections were stained by replacing the primary antibodies with PBS. Measurements were performed using the image analyzer program, Leica LAS X 2D s/w analysis and measurement modules (Leica Microsystems, Wetzlar, Germany) installed on a computer at the Histology Department, Faculty of Medicine, Ain Shams University, Cairo, Egypt. The computer was connected to a Leica DM2500 microscope with a built-in camera (Leica K3C camera). Five different randomly selected non-overlapping high-power fields (40x) were examined in each group to measure the mean number of degenerated neurons in H&E-stained sections, the mean area percentage of caspase-3 positive immune reaction, and the mean area percentage of GFAP positive immune reaction in astrocytes. The three parameters were measured in the prefrontal cortex, the CA1 hippocampal area, and the CA3 hippocampal area of all studied groups.

### 2.7 Statistical analysis

Statistical analyses were performed using GraphPad Prism software, version 8.0.1 (GraphPad Software Inc., San Diego, CA, United States; 2018). The Shapiro-Wilk normality test was applied to assess the distribution of data. All values were presented as the mean ± standard error of the mean (SEM). Comparisons among different experimental groups were conducted using a two-way analysis of variance (ANOVA) followed by Tukey’s multiple comparison *post hoc* test to determine specific group differences. A repeated-measures ANOVA was employed to analyze the body weight and the latency to reach the platform in the MWM test. A *p* value < 0.05 was considered statistically significant throughout the analyses.

## 3 Results

### 3.1 LURA mitigates DOX-induced body weight reduction

**FIGURE 2:**
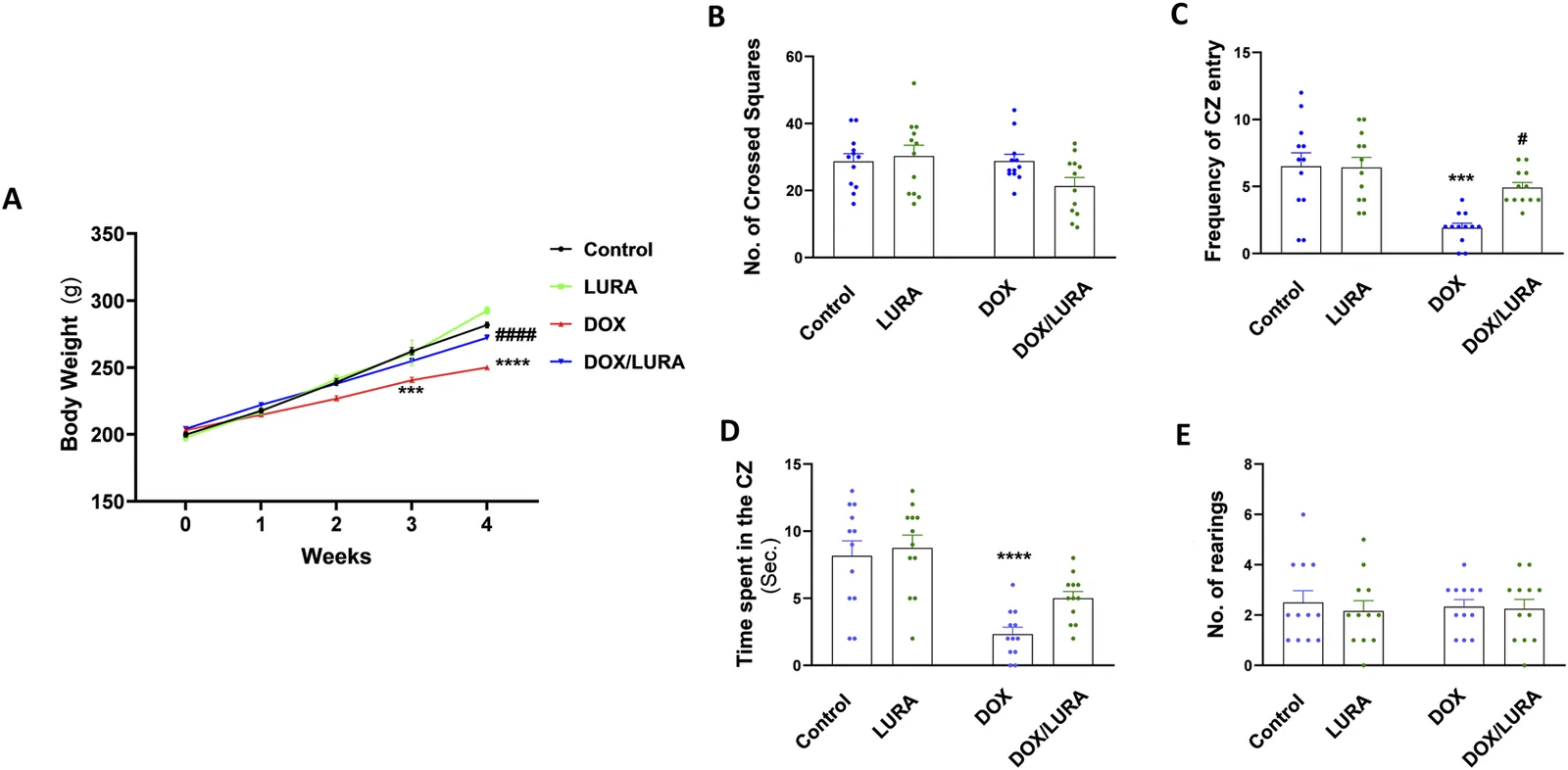
Effects of LURA on **(A)** body weight and **(B–E)** open field test behavioral parameters in DOX-treated rats. Data are expressed as mean ± S.E.M. (n = 12). ^***^ *p* < 0.001, ^****^ *p* < 0.0001 vs. the control group; ^#^ *p* < 0.05, ^####^ *p* < 0.0001 vs. the DOX group. Comparisons among groups were performed using two-way ANOVA followed by Tukey’s *post hoc* test, except for body weight, which was analyzed using repeated-measures ANOVA followed by Tukey’s *post hoc* test.

To assess the systemic toxicity of DOX over the course of the experiment and determine whether LURA can counteract its associated wasting effect, animal body weights were monitored weekly. As illustrated in Figure 2A, repeated-measures ANOVA revealed a significant main effect of DOX (F _(1, 44)_ = 12.56, *p* = 0.0009), LURA (F _(1, 44)_ = 7.66, *p* = 0.0082), and time (F _(4, 176)_ = 788.2, *p* < 0.0001). However, the interaction among DOX, LURA, and time was not statistically significant (F _(4,176)_ = 1.227, *p* = 0.3009). Treatment with DOX resulted in a marked reduction in body weight, which became significant by the end of the third week (*p* < 0.001) and persisted through the fourth week (*p* < 0.0001) when compared with the control group. Conversely, animals in the DOX/LURA group showed a significant improvement in body weight at the end of the fourth week (*p* < 0.0001) relative to the DOX-treated group. These results indicate that DOX causes progressive weight reduction, which is significantly alleviated by chronic LURA administration by the end of the treatment period.

### 3.2 Behavioral assessments

#### 3.2.1 LURA improves DOX-induced anxiety-like behavior in the open field test

To assess whether DOX induces locomotor deficits or anxiety-like behaviors, and to evaluate the potential protective effects of LURA, animals were subjected to the open field test. As illustrated in Figures 2B–E, two-way ANOVA indicated no significant interaction between DOX and LURA in the number of crossed squares, time spent in the CZ, or number of rearings (F _(1, 44)_ = 3.11, *p* = 0.085; F _(1, 44)_ = 1.616, *p* = 0.2104; F _(1, 44)_ = 0.1037, *p* = 0.749, respectively). However, a significant main effect was observed for the frequency of CZ entry (F _(1, 44)_ = 5.148, *p* = 0.0282). Compared with the control group, the DOX group exhibited a pronounced reduction in both the frequency of CZ entries (*p* < 0.001) and time spent in the CZ (*p* < 0.0001). Interestingly, the DOX/LURA group demonstrated a significant improvement in the frequency of CZ entries (*p* < 0.05) relative to the DOX group. These findings indicate that DOX did not impair the general locomotor activity; rather, it impairs exploratory drive and induces anxiety-like traits. Concomitant LURA treatment partially mitigates these behavioral deficits by restoring central zone exploration.

#### 3.2.2 LURA rescues DOX-induced spatial memory retention deficits in the Morris water maze

To evaluate the effect of LURA on DOX-induced spatial learning and memory deficits, we performed the Morris water maze test.

**FIGURE 3:**
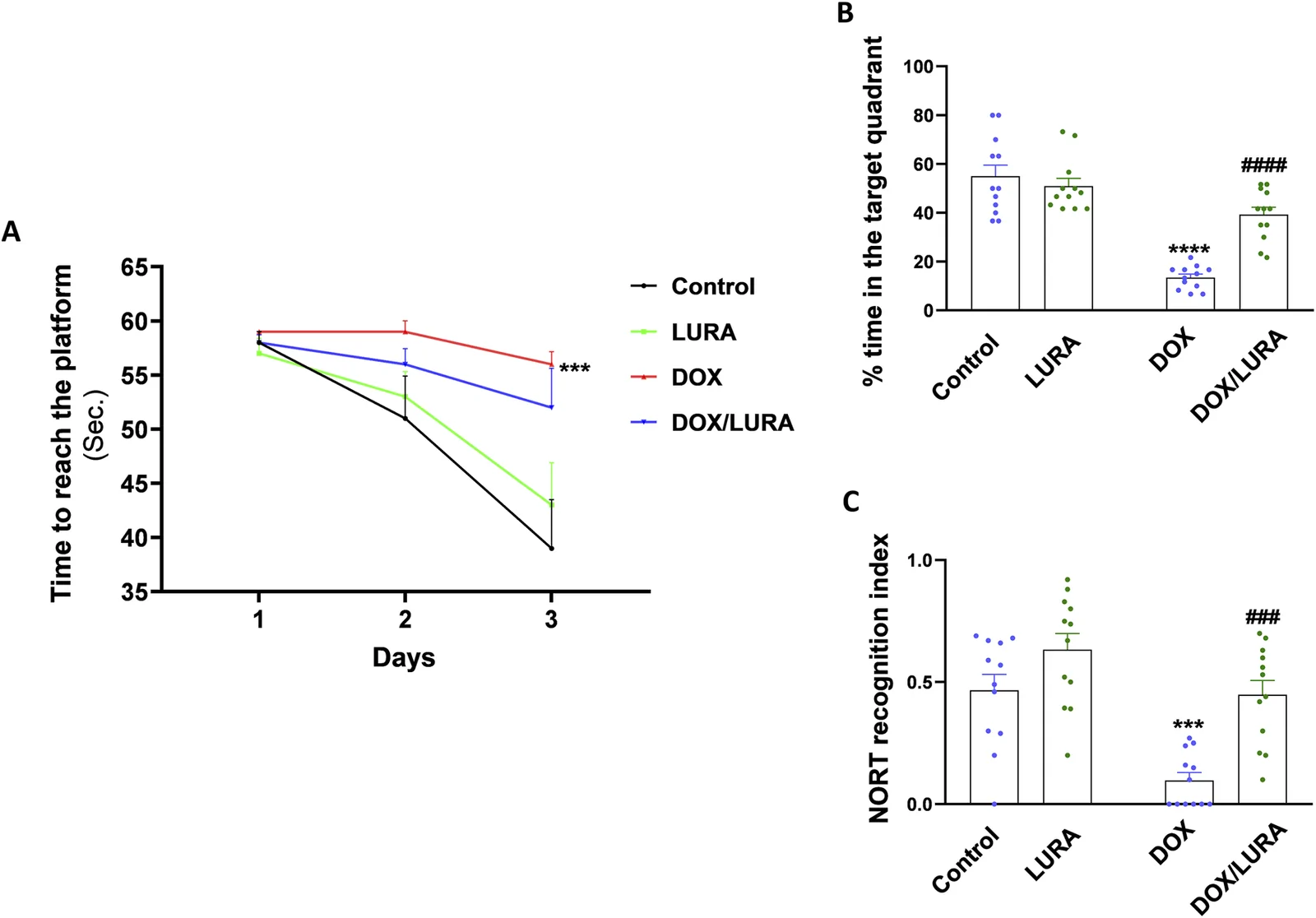
Effects of LURA on **(A,B)** MWM behavioral parameters and **(C)** NORT recognition index in DOX-treated rats. Data are expressed as mean ± S.E.M. (n = 12). ^***^ *p* < 0.001, ^****^ *p* < 0.0001 vs. the control group; ^###^ *p* < 0.001, ^####^ *p* < 0.0001 vs. the DOX group. Comparisons among groups were performed using two-way ANOVA followed by Tukey’s *post hoc* test, except for the time to reach the platform in the MWM, which was analyzed using repeated-measures ANOVA followed by Tukey’s *post hoc* test.

Regarding the time required to locate the hidden platform, repeated-measures ANOVA demonstrated a significant main effect of DOX (F _(1, 44)_ = 19.85, *p* < 0.0001) and time (F _(2, 88)_ = 18.10, *p* < 0.0001), whereas LURA treatment did not show a significant main effect (F _(1, 44)_ = 0.1174, *p* = 0.7324). No significant interaction among DOX, LURA, and time was observed (F _(2, 88)_ = 0.6394, *p* = 0.5292). As illustrated in Figure 3A, the DOX group exhibited a significant prolongation in the time to locate the platform on the third training day (*p* < 0.001) compared with the control group.

In the probe trial, two-way ANOVA revealed a significant interaction between DOX and LURA treatments (F _(1, 44)_ = 21.18, *p* < 0.0001). The DOX group spent a significantly lower percentage of time in the target quadrant (*p* < 0.0001) compared to the control group. Conversely, co-treatment with LURA significantly increased the percentage of time spent in the target quadrant (*p* < 0.0001) relative to the DOX group (Figure 3B).

Together, these results demonstrate that DOX severely impairs spatial memory retention rather than initial acquisition, an effect that is successfully reversed by LURA co-treatment.

#### 3.2.3 LURA mitigates DOX-induced recognition memory impairments in the novel object recognition test

To determine if DOX compromises short-term recognition memory and to assess the potential protective efficacy of LURA co-treatment, the novel object recognition test was conducted.

As shown in Figure 3C, two-way ANOVA revealed no significant interaction between DOX and LURA treatments (F _(1, 44)_ = 2.613, *p* = 0.113). The DOX group exhibited a marked reduction in the recognition index (*p* < 0.001) relative to the control group. Interestingly, LURA co-administration with DOX significantly increased the recognition index (*p* < 0.001) compared with the DOX group. These findings indicate that DOX significantly impairs object recognition memory, whereas LURA effectively protects against this cognitive deficit by restoring the animals’ preference for novelty.

### 3.3 Biochemical outcomes

#### 3.3.1 LURA reverses DOX-induced downregulation of *Glt-1* and *BDNF* gene expression in the hippocampus and prefrontal cortex

To explore the molecular mechanisms underlying DOX-induced cognitive impairment and assess if LURA exerts neuroprotective effects, we quantified the gene expression levels of *Glt-1* and *BDNF* in key cognitive brain regions.

**FIGURE 4:**
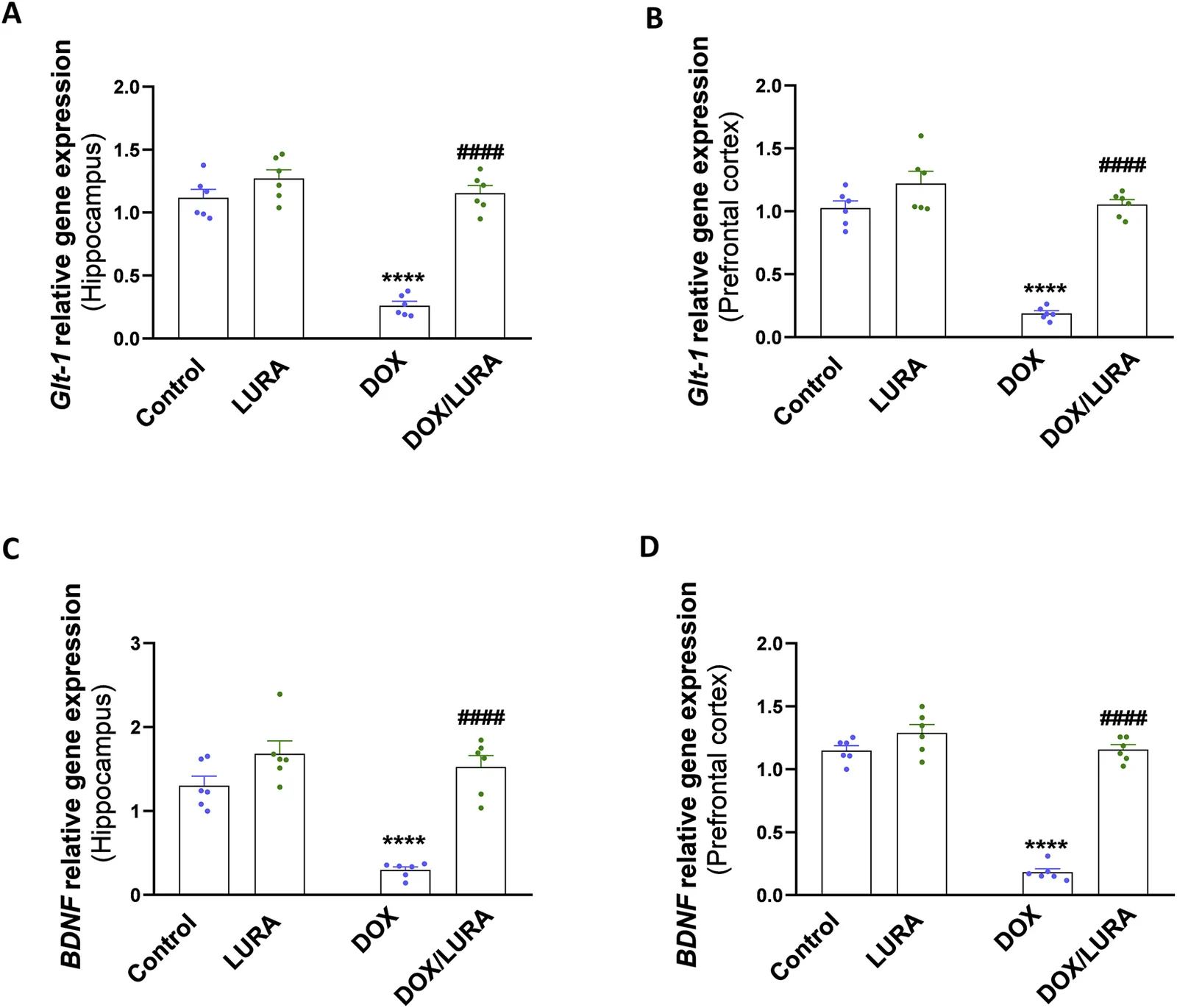
Effects of LURA on the relative gene expression levels of *Glt-1 *and *BDNF* in the hippocampus **(A, C)** and the prefrontal cortex **(B, D)** of DOX-treated rats. Data are expressed as mean ± S.E.M. (n = 6). ****p < 0.0001 vs. the control group; ^####^ *p* < 0.0001 vs. the DOX group. Comparisons among groups were performed using two-way ANOVA followed by Tukey’s post-hoc test.

As illustrated in Figure 4, two-way ANOVA revealed a significant interaction between DOX and LURA treatments for both *Glt-1* (F _(1,20)_ = 39.22, *p* < 0.0001 in the hippocampus; F _(1,20)_ = 31.18, *p* < 0.0001 in the prefrontal cortex) and *BDNF* (F _(1,20)_ = 13.18, *p* = 0.0017 in the hippocampus; F _(1,20)_ = 85.95, *p* < 0.0001 in the prefrontal cortex) gene expression levels. The DOX group showed a pronounced downregulation of *Glt-1* and *BDNF* expression in both brain regions (*p* < 0.0001) compared with the control group. Treatment with LURA markedly reversed these changes, resulting in a significant upregulation of both genes (*p* < 0.0001) *versus* the DOX group. These findings demonstrate that DOX suppresses critical markers of glutamate clearance and neuroplasticity in the hippocampus and prefrontal cortex, a molecular defect that is robustly rescued by LURA co-treatment.

#### 3.3.2 LURA restores protein levels of the CREB/BDNF/Glt-1 axis in the hippocampus and prefrontal cortex

To confirm whether the observed transcription changes translate to the functional protein level, we analyzed the protein expressions of CREB, p-CREB, BDNF, and Glt-1 in both the hippocampus and prefrontal cortex.

**FIGURE 5:**
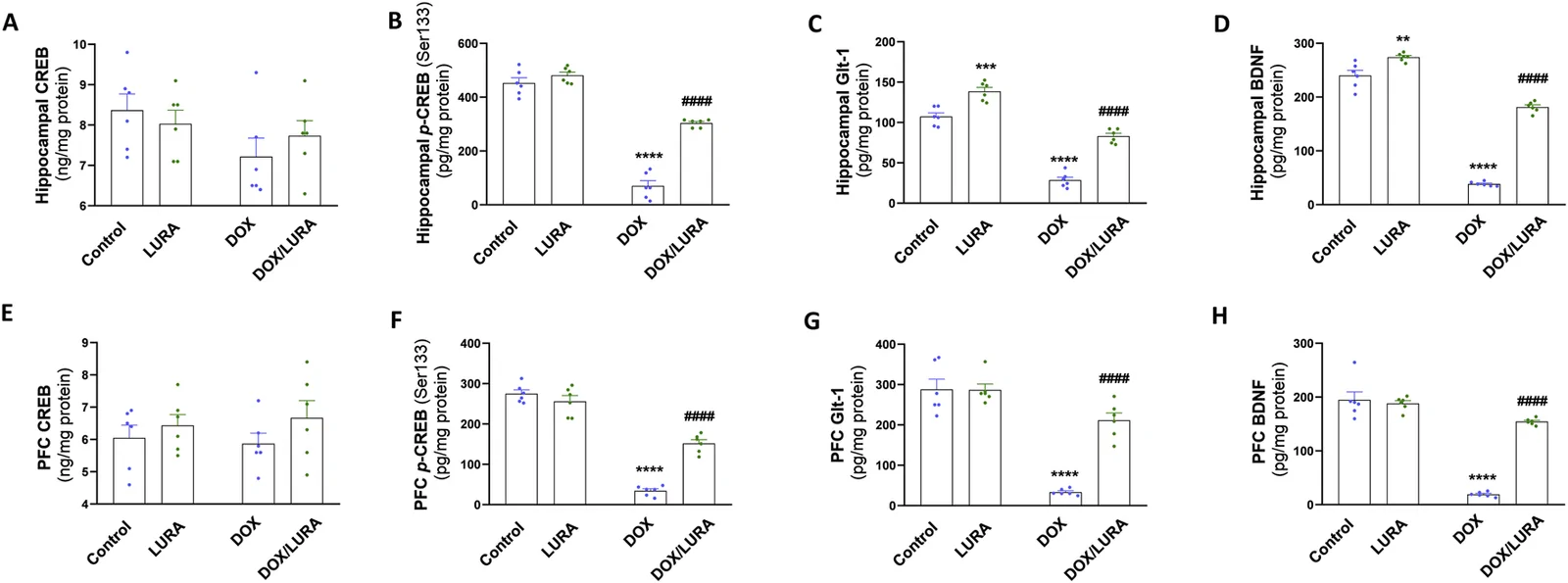
Effects of LURA on the protein levels of CREB, p-CREB, Glt-1, and BDNF in the hippocampus **(A–D)** and the prefrontal cortex **(E–H)** of DOX-treated rats. Data are expressed as mean ± S.E.M. (n = 6). **p < 0.01, ***p < 0.001, ****p < 0.0001 vs. the control group; ^####^ *p* < 0.0001 vs. the DOX group. Comparisons among groups were performed using two-way ANOVA followed by Tukey’s post-hoc test.

As shown in Figure 5, two-way ANOVA demonstrated a significant interaction between DOX and LURA treatments for p-CREB (F _(1,20)_ = 45.23, *p* < 0.0001; F _(1,20)_ = 46.19, *p* < 0.0001), Glt-1 (F _(1,20)_ = 7.595, *p* = 0.0122; F _(1,20)_ = 27.33, *p* < 0.0001), and BDNF (F _(1,20)_ = 99.68, *p* < 0.0001; F _(1,20)_ = 78.88, *p* < 0.0001) protein levels in the hippocampus and prefrontal cortex, respectively. Although the interaction was non-significant for the CREB protein level (F _(1,20)_ = 1.176, *p* = 0.291; F _(1,20)_ = 0.2623, *p* = 0.6141). The DOX group exhibited a significant reduction (*p* < 0.0001) in the levels of p-CREB, Glt-1, and BDNF in both brain regions compared to the control group, while CREB levels remained largely unaffected. Co-treatment of DOX and LURA significantly mitigated these reductions (*p* < 0.0001 vs. the DOX group). These protein-level findings validate our gene expression data, confirming that DOX suppresses the CREB/BDNF/Glt-1 axis, a pathological disruption that is functionally rescued by LURA.

#### 3.3.3 LURA restores the homeostasis of hippocampal and prefrontal GABA/glutamate balance disrupted by DOX

To investigate whether DOX induces disturbances in brain excitation/inhibition balance, and to evaluate if LURA can normalize this balance, we measured the concentrations of GABA and glutamate, as well as their ratio, in the hippocampus and prefrontal cortex.

**FIGURE 6:**
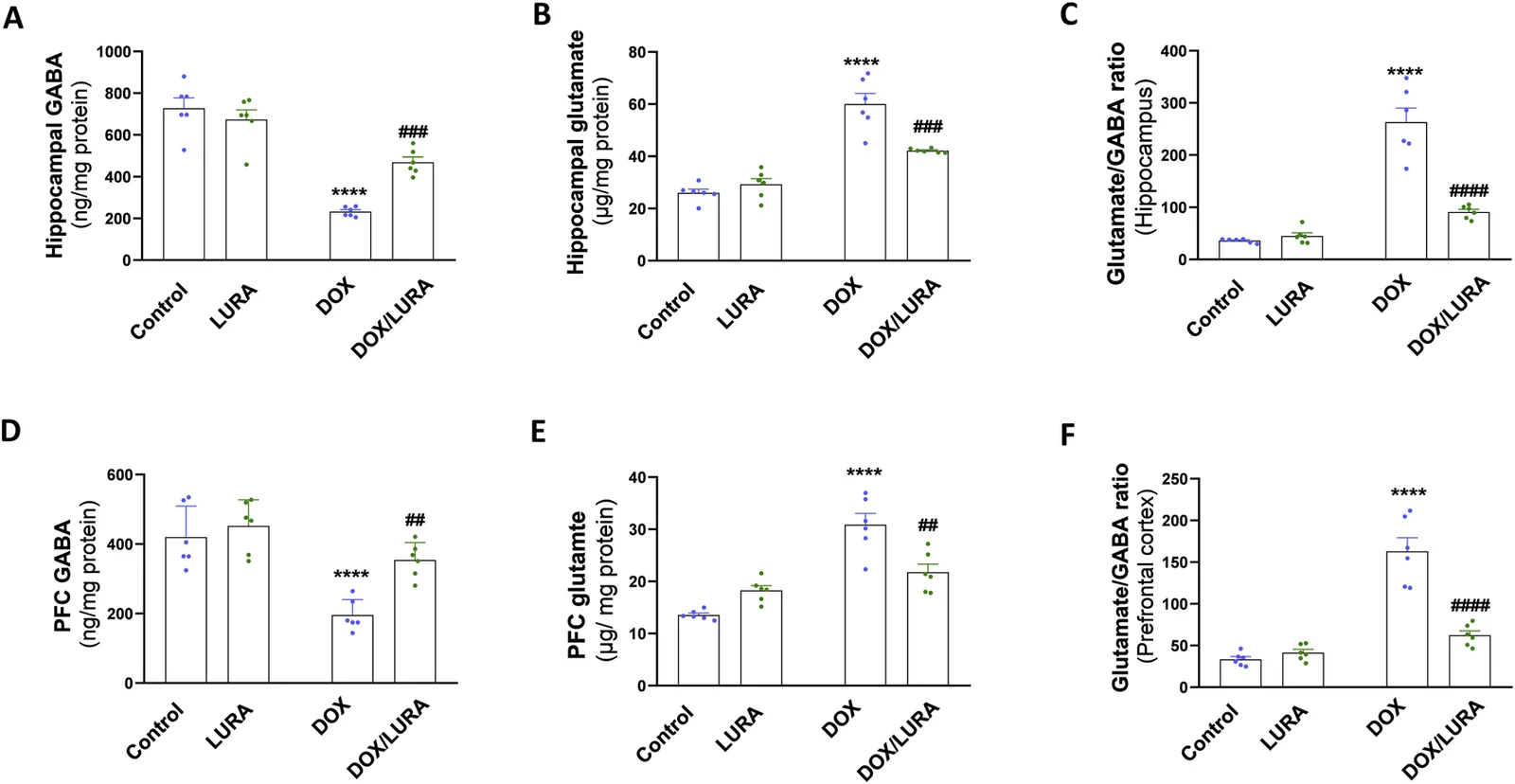
Effects of LURA on the GABA/glutamate balance in the hippocampus **(A–C)** and the prefrontal cortex **(D–F)** of DOX-treated rats. Data are expressed as mean ± S.E.M. (n = 6). ****p < 0.0001 vs. the control group; ^##^ *p* < 0.01, ^###^ *p* < 0.001, ^####^ *p* < 0.0001 vs. the DOX group. Comparisons among groups were performed using two-way ANOVA followed by Tukey’s post-hoc test.

As illustrated in Figure 6, two-way ANOVA revealed a significant interaction between DOX and LURA treatments for GABA (F _(1,20)_ = 16.44, *p* = 0.0006; F _(1,20)_ = 5.293, *p* = 0.0323), glutamate (F _(1,20)_ = 19.21, *p* = 0.0003; F _(1,20)_ = 23.63, *p* < 0.0001), and glutamate/GABA ratio (F _(1,20)_ = 41.21, *p* < 0.0001; F _(1,20)_ = 37.45, *p* < 0.0001) in the hippocampus and the prefrontal cortex, respectively. The DOX group displayed a marked reduction in GABA levels (*p* < 0.0001) accompanied by a significant increase in glutamate concentration and an elevated glutamate/GABA ratio (*p* < 0.0001) in both the hippocampus and prefrontal cortex compared with the control group. In contrast, DOX/LURA co-administration significantly restored the excitatory–inhibitory neurotransmitter balance by increasing GABA levels (*p* < 0.001 in the hippocampus; *p* < 0.01 in the prefrontal cortex) and decreasing glutamate levels (*p* < 0.001; *p* < 0.01), resulting in a substantial reduction in the glutamate/GABA ratio (*p* < 0.0001 in both regions) relative to the DOX group. These metrics demonstrate that DOX provokes an excitotoxic shift by driving up glutamate relative to GABA, a neurochemical imbalance that LURA effectively counteracts to re-establish a proper homeostatic tone.

#### 3.3.4 LURA suppresses DOX-induced oxidative and nitrosative stress in the hippocampus and prefrontal cortex

**FIGURE 7:**
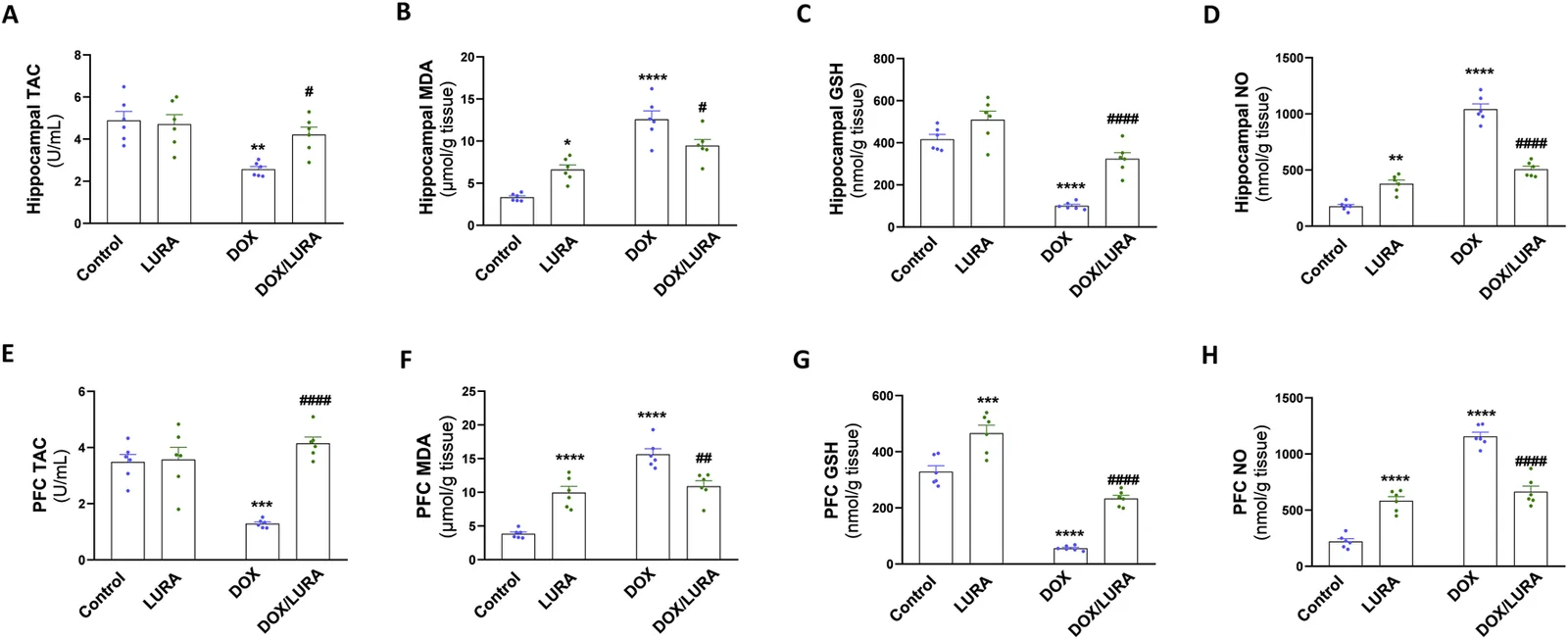
Effects of LURA on the markers of oxidative nitrosative stress in the hippocampus **(A–D)** and the prefrontal cortex **(E–H)** of DOX-treated rats. Data are expressed as mean ± S.E.M. (n = 6). *p < 0.05, **p < 0.01, ***p < 0.001, ****p < 0.0001 vs. the control group; ^#^ *p* < 0.05, ^##^ *p* < 0.01, ^####^ *p* < 0.0001 vs. the DOX group. Comparisons among groups were performed using two-way ANOVA followed by Tukey’s post-hoc test.

To evaluate whether LURA could rescue DOX-triggerred free radical damage and impairment of cellular antioxidant defenses, we measured biochemical markers of oxidative and nitrosative stress (TAC, GSH, MDA, and NO) in both brain areas. As presented in Figure 7, two-way ANOVA showed a significant interaction between DOX and LURA treatments for TAC (F _(1,20)_ = 6.338, *p* = 0.0205; F _(1,20)_ = 24.51, *p* < 0.0001), MDA (F _(1,20)_ = 21.5, *p* = 0.0002; F _(1,20)_ = 51.91, *p* < 0.0001), GSH (F _(1,20)_ = 5.65, *p* = 0.028; F _(1,20)_ = 1.159, *p* = 0.295), and NO (F _(1,20)_ = 127, *p* < 0.0001; F _(1,20)_ = 122.8, *p* < 0.0001) in the hippocampus and the prefrontal cortex, respectively. The DOX group exhibited a marked decrease in TAC (*p* < 0.01; *p* < 0.001) and GSH (*p* < 0.0001 in both regions) accompanied by a significant increase in MDA and NO (*p* < 0.0001 in both regions) relative to the control group. In contrast, the DOX/LURA group showed a significant increase in TAC (*p* < 0.05; *p* < 0.0001) and GSH (*p* < 0.0001 in both regions) together with a significant reduction in MDA (*p* < 0.05; *p* < 0.01) and NO (*p* < 0.0001 in both regions) compared to the DOX group. These findings indicate that DOX provokes robust central oxidative injury and nitrosative stress, a pathological burden that is effectively neutralized by the antioxidant properties of LURA co-treatment.

### 3.4 LURA preserves hippocampal and prefrontal neurostructural integrity and attenuates DOX-induced apoptosis and astrogliosis

To validate the neuroprotective potential of LURA against DOX-induced brain injury, we performed histopathological evaluations using H&E and toluidine blue staining, alongside immunohistochemical assessments for apoptotic (caspase-3) and astroglial activation (GFAP) markers.

**FIGURE 8:**
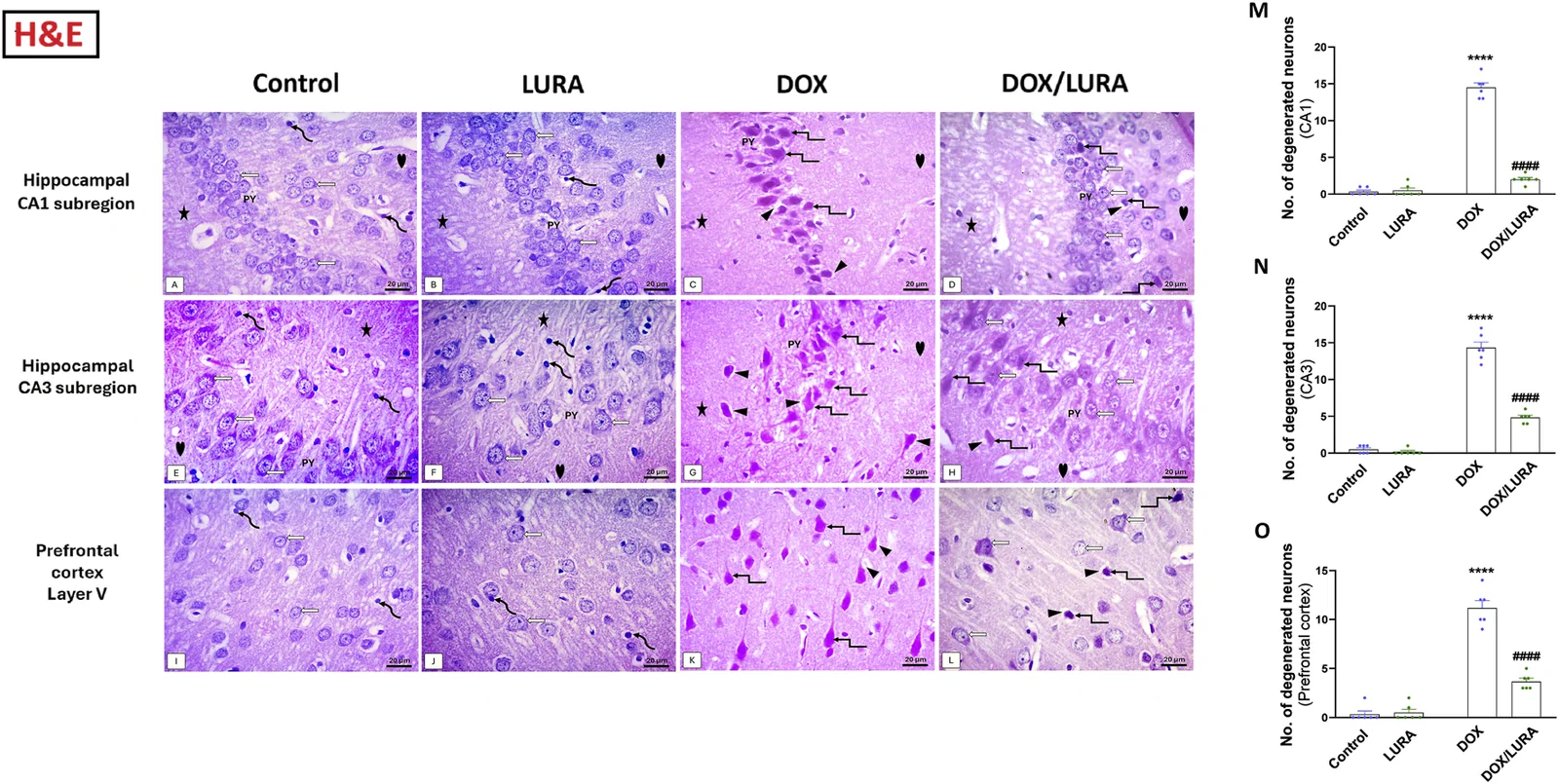
**(A–L)** Representative photomicrographs of H&E-stained sections of the hippocampal CA1 and CA3 areas and the prefrontal cortex. The control and LURA groups show viable neurons having vesicular nuclei and basophilic cytoplasm in the three areas (white arrows). The pyramidal cell layer of the hippocampus (PY) is located between the molecular (♥) and the polymorphic layers (asterisks). Some neuroglial cells are distributed between neurons, possessing small, round, deeply stained nuclei (curved arrows). Numerous damaged neurons are detected in the DOX group, having acidophilic cytoplasm and intensely stained pyknotic nuclei (elbow arrows); some of them appear shrunken and surrounded by widened perineuronal halos (▲). The DOX/LURA group exhibits multiple normal neurons (white arrows) and few scattered degenerated cells (elbow arrows). (x 400, scale bar: 20 µm). **(M–O)** Effects of LURA on the number of degenerated neurons in the hippocampal CA1 and CA3 areas and the prefrontal cortex of DOX-treated rats. Data are expressed as mean ± S.E.M. (n = 6). ^****^ *p* < 0.0001 vs. the control group; ^####^ *p* < 0.0001 vs. the DOX group. Comparisons among groups were performed using two-way ANOVA followed by Tukey’s *post hoc* test.

H&E-stained sections of the hippocampal CA1 and CA3 subfields and the prefrontal cortex (layer V) of the control group showed viable neurons possessing vesicular nuclei and basophilic cytoplasm. The pyramidal cell layer of the hippocampus was bordered by the molecular layer on one side and the polymorphic layer on the other side. Neuroglial cells were seen dispersed between neurons. A similar pattern was noticed in the LURA group. Sections of the DOX group revealed numerous degenerated neurons having acidophilic cytoplasm and intensely stained pyknotic nuclei. Some of them were shrunken and surrounded by wide perineuronal gaps. Examination of the sections of the DOX/LURA group showed nearly normal neuronal architecture; however, few degenerated cells were observed (Figure 8).

**FIGURE 9:**
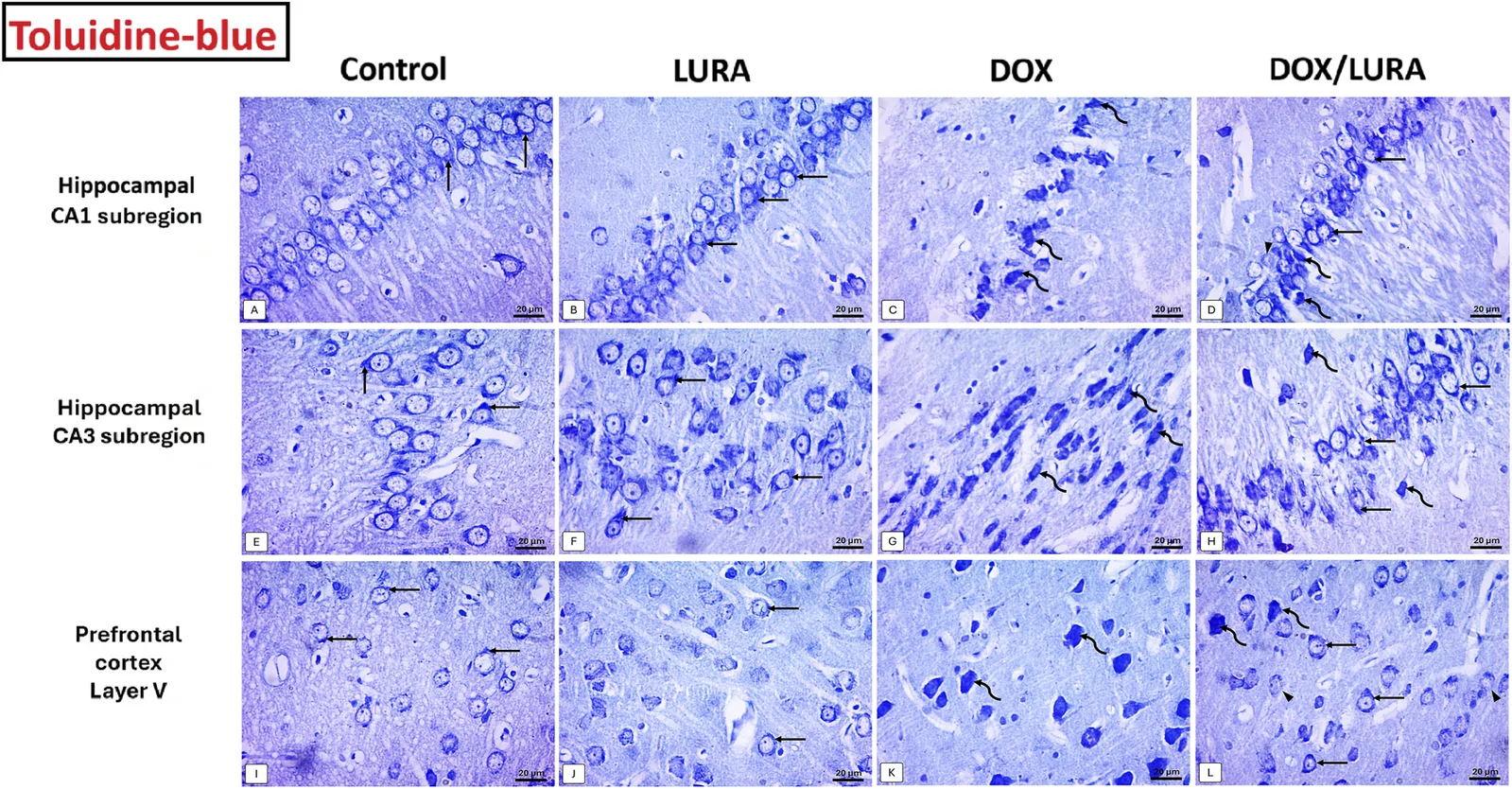
**(A–L)** Representative photomicrographs of toluidine blue-stained sections of the hippocampal CA1 and CA3 areas and the prefrontal cortex. The control and LURA groups exhibit normal neurons studied with clusters of Nissl’s granules (↑). The DOX group reveals numerous deeply stained distorted neurons (curved arrows). The DOX/LURA group shows restoration of the normal pattern of most neurons; few deeply stained neurons still exist. Neurons with an apparent decrease in Nissl’s granules (▲) can be detected (×400, scale bar: 20 µm).

Toluidine blue-stained sections demonstrated numerous viable neurons containing evident clumps of Nissl’s bodies surrounding the vesicular nuclei in the three brain areas of both the control and the LURA groups. Whereas examination of the DOX group sections revealed distorted neurons. Most neurons appeared deeply stained. The nuclei were indistinguishable from the perikaryon. In the DOX/LURA group nearly normal neurons were frequently seen. Few neurons with an apparent decrease in Nissl’s granules were detected. Deeply stained neurons were occasionally observed (Figure 9).

**FIGURE 10:**
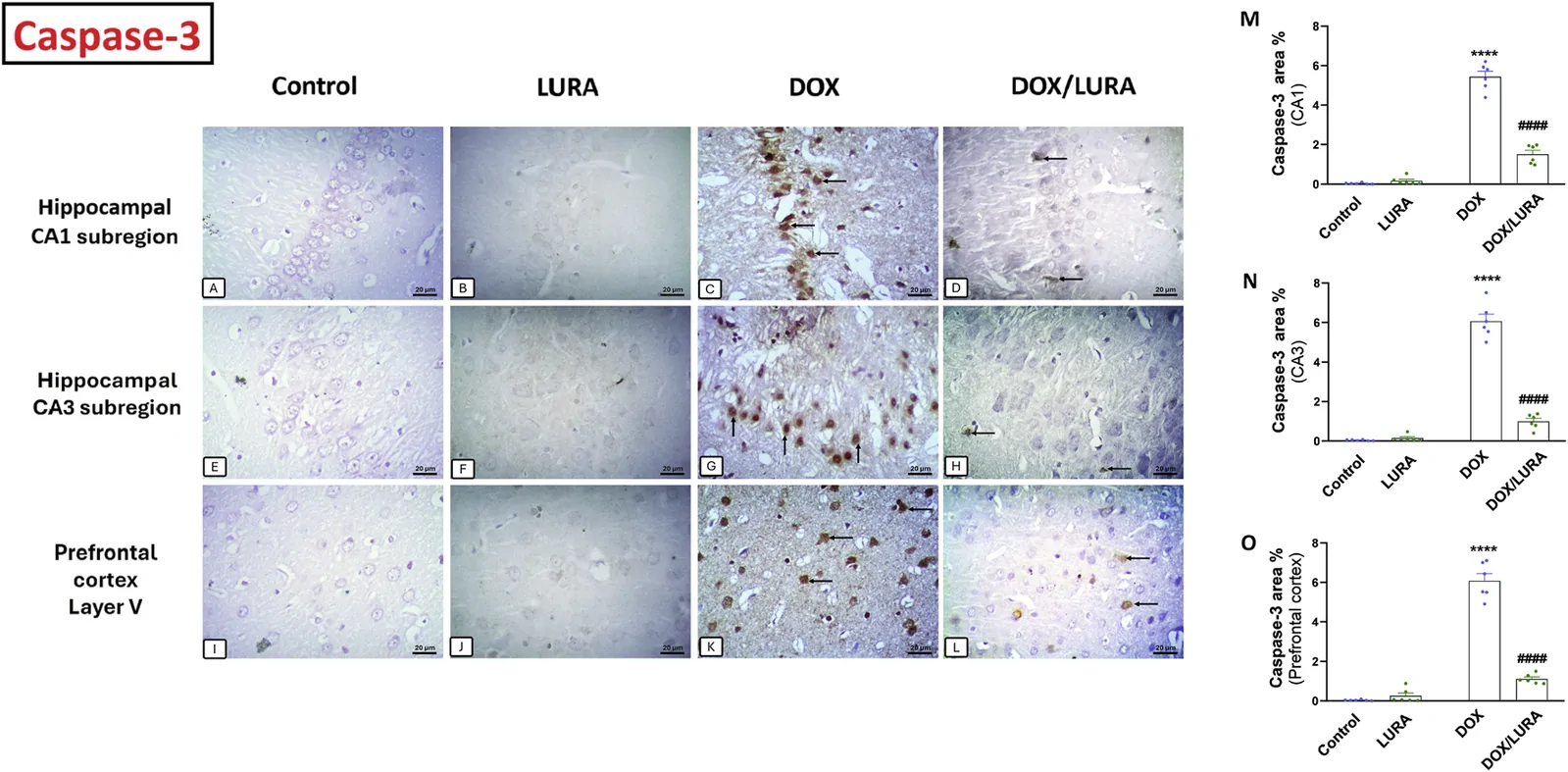
**(A–L)** Representative photomicrographs showing anti-caspase-3 immunoreactivity in the hippocampal CA1 and CA3 areas and the prefrontal cortex. The control and LURA groups exhibit a slight faint reaction in very few neurons. Most of the neurons in the DOX group show a marked increase in both cytoplasmic and nuclear caspase-3 immune reaction (black arrows). The DOX/LURA group reveals few immunoreactive neurons (↑). (avidin–biotin peroxidase ×400, scale bar: 20 µm). **(M–O)** Effects of LURA on the mean area % of caspase-3 positive immune reaction in the hippocampal CA1 and CA3 areas and the prefrontal cortex of DOX-treated rats. Data are expressed as mean ± S.E.M. (n = 6). ^****^ *p* < 0.0001 vs. the control group; ^####^ *p* < 0.0001 vs. the DOX group. Comparisons among groups were performed using two-way ANOVA followed by Tukey’s *post hoc* test.

**FIGURE 11:**
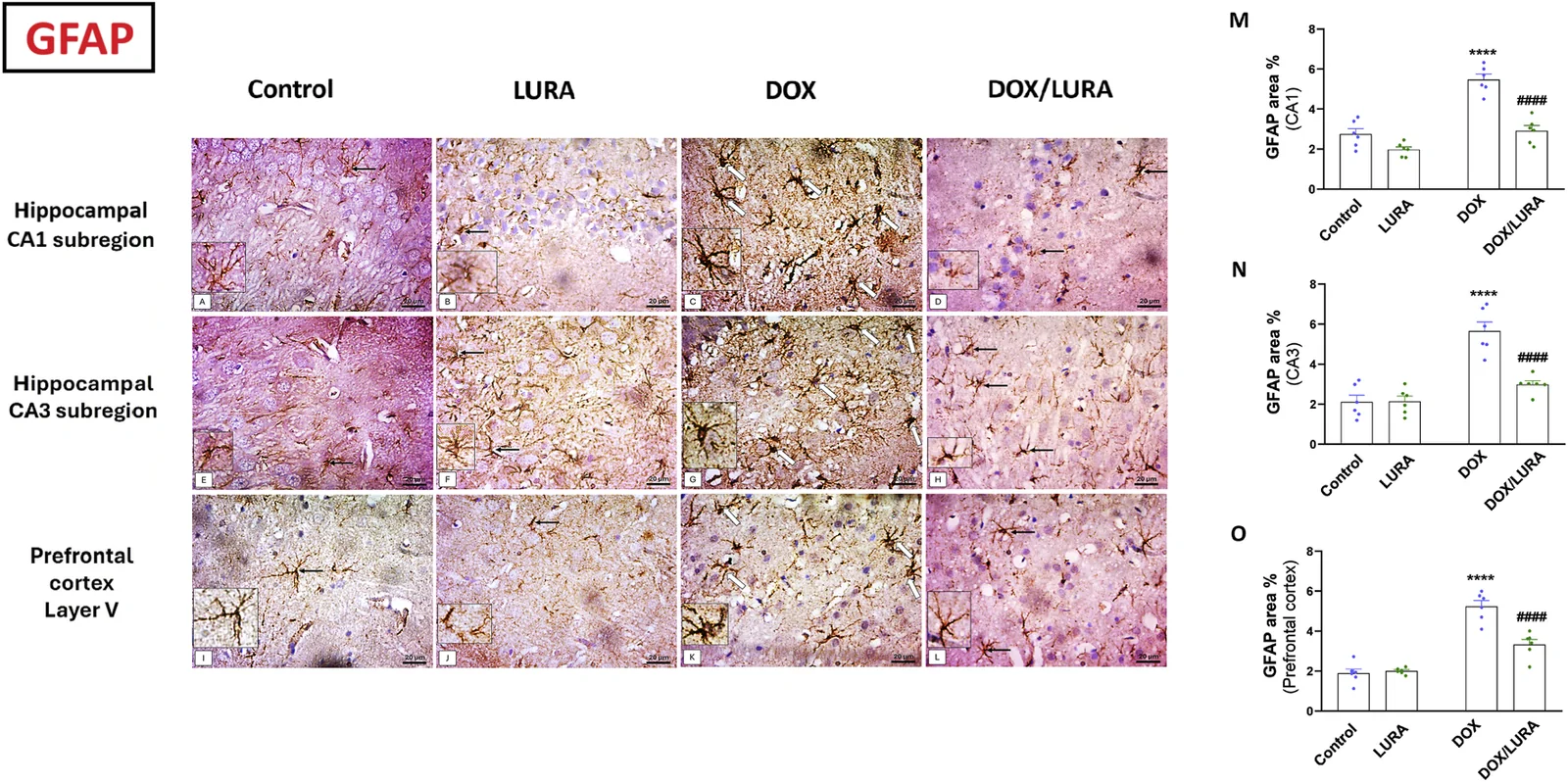
**(A-L)** Representative photomicrographs showing anti-GFAP immunoreactivity in the hippocampal CA1 and CA3 areas and the prefrontal cortex. The control and LURA groups show regular cytoplasmic GFAP reaction in astrocytes and their processes (black arrows). Increased GFAP reaction is seen in numerous astrocytes (white arrows) in the DOX group. The DOX/LURA group reveals a moderate astrocytic GFAP reaction (black arrows). (avidin–biotin peroxidase ×400, insets ×1,000, scale bar: 20 µm). **(M-O)** Effects of LURA on the mean area % of GFAP positive immune reaction in the hippocampal CA1 and CA3 areas and the prefrontal cortex of DOX-treated rats. Data are expressed as mean ± S.E.M. (n = 6). ^****^ *p* < 0.0001 vs. the control group; ^####^ *p* < 0.0001 vs. the DOX group. Comparisons among groups were performed using two-way ANOVA followed by Tukey’s *post hoc* test.

In the anti-caspase-3-stained sections, a weak immune reaction was observed in very few neurons of the control and LURA groups. An intense cytoplasmic and nuclear positive reaction was seen in most neurons of the DOX group. The sections of the DOX/LURA group exhibited a weak anti-caspase-3 immune reaction. Similarly, anti-GFAP-stained sections of the control and LURA groups showed positive cytoplasmic GFAP expression in the astrocytes and their cytoplasmic processes. The DOX group exhibited increased GFAP immunoreactivity with apparent astrocytic hypertrophy compared to the control group. This was reflected by a significant increase in the area percentage of GFAP-positive staining, consistent with astrocytic activation. A weak positive GFAP cytoplasmic expression was shown by the DOX/LURA group (Figures 10, 11).

Regarding the quantitative histological and immunohistochemical analyses (Figures 8, 10, 11; Supplementary Table S1), the DOX group exhibited a significant increase in the number of degenerated neurons and the caspase-3 and GFAP immunoreactivities (*p* < 0.0001) compared to the control group in the three tested areas. This was improved in the DOX/LURA group, which exhibited significant decreases in the three outcomes in the three tested areas (*p* < 0.001 to *p* < 0.0001). In summary, these microscopic findings confirm that DOX causes severe cellular degeneration, apoptosis, and reactive astrogliosis within key cognitive brain structures, all of which are profoundly suppressed and structurally normalized by LURA co-treatment.

## 4 Discussion

Emerging evidence indicates that cognitive impairment, commonly known as “chemobrain”, affects up to 70% of cancer survivors who have undergone chemotherapy, spanning multiple domains such as memory, attention, and executive function. Over the years, chemobrain has been prevalent among cancer survivors whose predominant chemotherapeutic treatment was doxorubicin (John et al., 2021; Ongnok et al., 2020).

In the current study, DOX administration (2.5 mg/kg/week for 4 weeks) recapitulated the core behavioral phenotype of CICI, reducing the exploration behavior and increasing anxiety-like behavior as denoted by the reduced open field central zone entries, disrupting the spatial memory as depicted in the diminished target quadrant preference in the Morris water maze, and impairing the non-spatial recognition memory as illustrated by the lowered novel object recognition index. Similar results were obtained in previous studies, where DOX administration resulted in comparable behavioral impairments (Alhowail and Aldubayan, 2023; Aboul-Fotouh et al., 2026). Such deficits mirror clinical reports of chemotherapy-exposed cancer survivors, where deficits in delayed memory and cognitive impairment were reported in patients with breast cancer following the administration of doxorubicin and cyclophosphamide (Ramalho et al., 2017; Andryszak et al., 2018).

These detected behavioral impairments align closely with the hippocampal-prefrontal dysfunction reported in our results, where DOX elevated glutamate levels while depleting GABA, yielding markedly higher glutamate/GABA ratios in both regions, a neurochemical hallmark of excitotoxicity. This imbalance is consistent with and may contribute to the observed cognitive decline where excessive glutamatergic transmission disrupts synaptic plasticity essential for spatial learning and object discrimination, while GABAergic deficits exacerbate anxiety-like behavior. This imbalance had been mechanistically explained by Thomas and colleagues in their study, where they reported that DOX administration induced disruption of glutamate handling in the synaptic cleft through reduction in glutamate clearance secondary to lowering the glutamate uptake rate constant in the frontal cortex of mice. The authors further suggested that this slower clearance may reflect reduced expression of glial glutamate transporters or increased glutamate release from glial cells, particularly astrocytes (Thomas et al., 2017).

Indeed, our data further support the hypothesis outline above, where the present study identified a substantial downregulation of glutamate transporter-1 (Glt-1), the primary astrocytic protein responsible for approximately 90% of glutamate clearance from the synaptic cleft (Pekny and Pekna, 2014), providing the cellular mechanism behind the impairment of glutamate clearance. The loss of Glt-1 functionality, likely driven by DOX-induced oxidative stress and inflammatory cascades involving TNF-α (Zou and Crews, 2005), offers a candidate cellular explanation for the observed glutamate overflow that would give rise to a cycle of oxidative damage, mitochondrial dysfunction, and neuronal apoptosis.

Lurasidone, the atypical antipsychotic, co-treatment comprehensively ameliorated the cognitive performance, normalizing probe trial quadrant preference, recognition index, and central zone exploration. These outcomes not only represent simple behavioral improvements but also reflect systems-level repair as clearly illustrated by the optimization of glutamate/GABA ratios through Glt-1 upregulation, restoring astrocytic uptake capacity and preventing excitotoxic cascades that drive DOX-impaired cognitive domains. This LURA-induced glutamate modulation is already in line with recent evidence that the drug attenuates NMDA-receptor-antagonist-induced glutamatergic hyperactivity (Okada et al., 2019) and reduces astroglia glutamate release *via* 5-HT_7_ receptor-mediated mechanisms (Fukuyama et al., 2022), as well as normalizes GABAergic transmission in the hippocampus of mice (Luoni et al., 2017).

Although our data indicate that LURA normalizes glutamatergic hyperactivity and attenuates conditions favoring excitotoxicity, concerns have been raised that developmental exposure to this drug may disrupt excitatory-inhibitory balance in a different context, contributing to neurodevelopmental behavioral impairment, as observed in zebrafish embryos (Li et al., 2025). However, a recent large multinational population-based cohort study suggested there is little to no increased risk of child neurodevelopmental disorders or learning difficulties after prenatal exposure to antipsychotics, including lurasidone (Bruno et al., 2024).

Concomitantly, DOX elicited a pronounced reduction in CREB phosphorylation alongside diminished BDNF expression in the hippocampus and prefrontal cortex, abolishing neurotrophic support for neuronal survival and dendritic remodeling critical to memory consolidation. Mechanistically, CREB transcriptional activity depends on upstream kinase cascades, including ERK/MAPK, CaMKII, and PKA, that are themselves sensitive to disruption by sustained oxidative stress and inflammatory signaling (Wang and Mao, 2019). The marked oxidative/nitrosative burden documented in our DOX-treated animals, as manifested by the reduced TAC/GSH and elevated MDA/NO, therefore offers one plausible route by which p-CREB, and consequently BDNF transcription, may have been suppressed. A second contributor may be the glutamate excess itself, since sustained overactivation of glutamate receptors is a recognized trigger of excitotoxic calcium dysregulation capable of impairing neurotrophic signaling (Nicosia et al., 2024), raising the possibility that glutamate overflow and CREB/BDNF suppression are mutually reinforcing rather than two independent findings. Behaviorally, this manifests as the multi-domain deficits observed in our results, paralleling BDNF/TrkB reductions linked to hippocampal neurogenesis loss in prior DOX models (Park et al., 2018).

Histologically, CA1/CA3 pyramidal degeneration, distorted neurons with Nissl’s granules loss, and caspase-3 immunoreactivity confirm downstream neurodegeneration, while GFAP upregulation signals reactive gliosis; both are structural hallmarks linking molecular dysregulation to functional loss observed in DOX-administered rats. Simultaneously, LURA co-administration, in a dose of 3 mg/kg for 4 weeks, reinstated p-CREB, BDNF, and Glt-1 expression across the prefrontal cortex and hippocampus, restoring neuroplasticity pathways compromised by DOX.

The LURA-induced spatial and recognition memory recovery aligns with this targeted neurotrophic rescue, as CREB/BDNF signaling governs hippocampal-dependent tasks. Histological preservation of neuronal architecture, as depicted in the viable CA1/CA3 morphology and preserved Nissl bodies along with the blunted caspase-3 activation, provides anatomical validation, highlighting LURA as a pleiotropic agent that stabilizes glutamate signaling and promotes neuronal survival. This LURA-induced neurotrophic rescue, probably attributed to its modulation of 5HTR-7 receptors, was reported in literature, where the chronic administration of the drug was able to normalize BDNF/CREB signaling that was otherwise downregulated in chronic mild stress models (Luoni et al., 2015; Begni et al., 2024). The functional relevance of Glt-1 restoration to cognition is reported in a different yet relevant context, a temporal lobe epilepsy model study, in which pharmacological Glt-1 upregulation was sufficient to reduce hippocampal glutamate and improve performance on the same two cognitive tasks used here, the Morris water maze and novel object recognition (Ramandi et al., 2021). The present study establishes that Glt-1 restoration is mechanistically capable of driving the glutamate normalization and cognitive improvement observed here alongside LURA’s effect on Glt-1.

The detrimental effect of DOX on oxidative/nitrosative stress can further explain the above findings. Although DOX is well-known for its poor BBB penetration (Ongnok et al., 2020), it clearly resulted in brain oxidative/nitrosative damage in the current study, depleting total antioxidant capacity (TAC) and glutathione (GSH) while surging malondialdehyde (MDA) and nitric oxide (NO), a context potentiating the observed excitotoxicity, apoptosis, and gliosis. Indeed, the present results can further be explained by the well-established DOX-induced peripheral oxidative stress and inflammation related to the generation of reactive oxygen species (ROS) (Keeney et al., 2018; Ongnok et al., 2020), initiating a cascade of oxidative/nitrosative damage matching our reduced TAC/GSH levels and upregulated MDA/NO findings.

These redox shifts are plausibly capable of exacerbating glutamate overflow by impairing astrocytic Glt-1 function and CREB activation and may therefore have contributed to the memory deficits observed. In fact, it has been reported that oxidative stress causes indirect alterations in glutamate clearance, where receptor-mediated alterations in glutamate uptake through NF-kB have been demonstrated (Zou and Crews, 2005). Additionally, TNF has also been shown to inhibit EAAT2, the human homolog of GLT-1, in primary normal human fetal astrocytes by interacting with transcription and EAAT2 promoter activity as well as decreasing mRNA and protein levels (Su et al., 2003).

LURA interrupted this redox cascade by elevating TAC/GSH levels while suppressing MDA/NO surges, yielding robust behavioral recovery alongside preserved histological architecture, evidenced by reduced neuronal shrinkage and minimized perineuronal spacing. This aligns with the previously reported LURA’s ability to rescue the impairment in antioxidant responsiveness in both the hippocampus and prefrontal cortex of rats exposed to chronic restraint stress (Spero et al., 2022). LURA’s antioxidant effects likely arise from multimodal serotonin receptor modulation, upregulating BDNF alongside antioxidant enzyme expression. 5-HT_2A_ blockade has been reported to reduce oxidative stress while enhancing antioxidant enzyme availability (Kaur and Krishan, 2020), while partial 5-HT_1A_ agonism transcriptionally induces antioxidant genes (Biswal et al., 2015). Moreover, the drug’s 5-HT_7_ antagonism further contributes to the previously mentioned normalization of BDNF (Begni et al., 2024). This BDNF-redox axis offers a possible pharmacological link between our observed TAC/GSH restoration and LURA’s broader neuroprotective actions, although the present, correlative data do not establish this link directly.

While other agents have been explored for DOX-induced chemobrain, LURA offers unique advantages. Unlike the acetylcholinesterase inhibitor donepezil, which primarily targets the cholinergic system (Ongnok et al., 2021), LURA addresses the glutamatergic dysregulation that is increasingly recognized as a core driver of DOX neurotoxicity. Moreover, while antioxidants like resveratrol can mitigate oxidative stress (Moretti et al., 2021), they may not fully address the underlying synaptic architectural damage. LURA, by contrast, not only reduced markers of oxidative stress, MDA and NO, and increased antioxidant capacity, TAC and GSH, but also preserved nearly normal neuronal architecture.

Collectively, the current study provides multilevel findings that underline a unified mechanism where DOX triggers oxidative/nitrosative stress and glial dysfunction, downregulating CREB/BDNF/Glt-1, triggering glutamate/GABA imbalance, neurodegeneration, and domain-specific cognitive impairment. Our results reveal that lurasidone powerfully mitigates DOX-induced cognitive impairment in rats through coordinated restoration of hippocampal and prefrontal glutamate homeostasis, CREB/BDNF/Glt-1 signaling, redox balance, and structural integrity, as evidenced by normalized behavioral performance, glutamate/GABA ratios, protein expression, oxidative markers, and histopathology across both brain regions, the hippocampus and prefrontal cortex.

## 5 Limitations

This study has multiple limitations that should be considered when interpreting its findings. First, our experimental design utilized a single, fixed dose of LURA based on previously published literature showing efficacy in neuropharmacological rat models. Future studies evaluating a multi-dose escalation matrix are required to fully characterize the pharmacological profile of LURA in this specific chemobrain context. Second, this study was conducted exclusively on male Wistar rats to minimize the confounding variations introduced by the estrous cycle on behavioral assessments and baseline molecular signaling. However, CICI is highly prevalent in female patient populations (e.g., breast cancer survivors undergoing DOX regimens). Subsequent preclinical trials incorporating female cohorts are essential to address potential sex-dependent dimorphisms in LURA’s therapeutic response. Third, although our behavioral battery successfully demonstrated improvements in recognition memory and spatial learning/memory, it captures only a subset of cognitive domains. Chemobrain clinically manifests as a multifaceted syndrome affecting a wide range of cognitive domains. Incorporating a broader array of behavioral assays (such as the attentional set-shifting task and the radial arm maze) would provide a more comprehensive characterization of LURA’s cognitive profile. Finally, further studies utilizing targeted pharmacological interventions (such as the selective 5-HT_7_ antagonist SB-269970, the TrkB inhibitor ANA-12, or the selective GLT-1 blocker dihydrokainate) as well as cell-specific genetic knockdown models could be important to further dissect the molecular mechanism of action of LURA in chemobrain.

## Data Availability

The raw data supporting the conclusions of this article will be made available by the authors, without undue reservation.
