# Supplementary material for: Lurasidone ameliorates doxorubicin-induced chemobrain in male Wistar rats: involvement of the hippocampal and prefrontal cortex CREB/BDNF/Glt-1 axis and glutamate homeostasis
Source: Front Pharmacol. 2026 Sep 9;17:1901510. doi: 10.3389/fphar.2026.1901510 (PMC13597779; doi:10.3389/fphar.2026.1901510)
Supplement: Supplementary file 1 [file Table1.docx]

**Lurasidone ameliorates doxorubicin-induced chemobrain in male Wistar rats: Involvement of the hippocampal and prefrontal cortex CREB/BDNF/Glt-1 axis and glutamate homeostasis**

Mohamed Z. Habib, Sherif A. Kamar, Mohamed Atef Elkholy, Yasmin M. Aboul-Ela, Mohamed Mahmoud Abdelrahim Elshaer, Nourhan Moussa, Nouran K. Olama, Sara Elsayed Abdelrahman, Mohammed R. Rabei, Ahmed A Salama, Esraa M. Elnahas

**Supplementary Table 1: Effects of LURA on the number of degenerated neurons and the mean area % of caspase-3 and GFAP positive immune reaction in the hippocampal CA1 and CA3 areas and the prefrontal cortex of DOX-treated rats**

|  |  | **Control** | **LURA** | **DOX** | **DOX/LURA** | **Two-way ANOVA** |  |
| --- | --- | --- | --- | --- | --- | --- | --- |
|  |  |  |  |  |  | **DOX – LURA treatments interaction** | |
| **Degenerated neurons**  (No.) | **CA1** | 0.33 ± 0.21 | 0.5 ± 0.34 | 14.5 ± 0.62^****^ | 2 ± 0.26^####^ | F _(1, 20)_ = 262.5, *p*<0.0001 | |
|  | **CA3** | 0.5 ± 0.22 | 0.17 ± 0.17 | 14.33 ± 0.76^****^ | 4.83 ± 0.31^####^ | F _(1, 20)_ = 112, *p*<0.0001 | |
|  | **Prefrontal Cortex** | 0.33 ± 0.33 | 0.5 ± 0.34 | 11.17 ± 0.75^****^ | 3.67 ± 0.33^####^ | F _(1, 20)_ = 65.31, *p*<0.0001 | |
| **Caspase-3**  (mean area %) | **CA1** | 0.03 ± 0.02 | 0.16 ± 0.08 | 5.44 ± 0.28^****^ | 1.51 ± 0.19^####^ | F _(1, 20)_ = 136.3, *p*<0.0001 | |
|  | **CA3** | 0.05 ± 0.01 | 0.15 ± 0.07 | 6.07 ± 0.36^****^ | 0.99 ± 0.15^####^ | F _(1, 20)_ = 174.4, *p*<0.0001 | |
|  | **Prefrontal Cortex** | 0.04 ± 0.01 | 0.26 ± 0.14 | 6.08 ± 0.37^****^ | 1.11 ± 0.1^####^ | F _(1, 20)_ = 162.8, *p*<0.0001 | |
| **GFAP**  (mean area %) | **CA1** | 2.75 ± 0.27 | 1.98 ± 0.14 | 5.48 ± 0.27^****^ | 2.92 ± 0.26^####^ | F _(1, 20)_ = 13.7, *p =* 0.0014 | |
|  | **CA3** | 2.12 ± 0.33 | 2.14 ± 0.26 | 5.66 ± 0.45^****^ | 2.99 ± 0.18^####^ | F _(1, 20)_ = 17.36, *p =* 0.0005 | |
|  | **Prefrontal Cortex** | 1.9 ± 0.21 | 2.01 ± 0.06 | 5.23 ± 0.3^****^ | 3.33 ± 0.26^###^ | F _(1, 20)_ = 20.33, *p =* 0.0002 | |

Data are expressed as mean ± S.E.M. (n = 6). **^****^***p* < 0.0001 vs. the control group; **^####^***p* < 0.0001 vs. the DOX group. Comparisons among groups were performed using two-way ANOVA followed by Tukey’s post-hoc test.
